# Exploring novel determinants of exercise behavior: a lagged exposure-wide approach

**DOI:** 10.1093/abm/kaae082

**Published:** 2025-01-04

**Authors:** Harold H Lee, Eric S Kim, Younseo Kim, David E Conroy, Tyler J VanderWeele

**Affiliations:** Department of Biobehavioral Health, The Pennsylvania State University, University Park, PA 16802, United States; Department of Psychology, University of British Columbia, BC V6T 1Z4, United States; Lee Kum Sheung Center for Health and Happiness, Harvard T.H. Chan School of Public Health, Boston, MA 02115, United States; Human Flourishing Program, Institute for Quantitative Social Science, Harvard University, Cambridge, MA 02138, United States; Department of Statistics, The Pennsylvania State University, University Park, PA 16802, United States; Department of Kinesiology, University of Michigan, Ann Arbor, MI 48109, United States; Human Flourishing Program, Institute for Quantitative Social Science, Harvard University, Cambridge, MA 02138, United States; Department of Epidemiology, Harvard T.H. Chan School of Public Health, Boston, MA 02115, United States; Department of Biostatistics, Harvard T.H. Chan School of Public Health, Boston, MA 02115, United States

**Keywords:** exercise, physical activity, lagged exposure-wide analysis, midlife

## Abstract

Many middle-aged to older adults do not engage in regular exercise at all, despite its importance for healthy aging. Extensive research grounded in behavioral and social science theories has identified numerous determinants of exercise. However, few studies used an exposure-wide approach, a data-driven exploratory method particularly useful for identifying novel determinants.

Methods: We used data from 13 771 participants in the Health and Retirement Study, a diverse, national panel study of adults aged >50 years in the United States, to evaluate 62 candidate determinants of exercise participation. Candidate predictors were drawn from the following domains: health behaviors, physical health, psychological well-being, psychological distress, social factors, and work. We used Poisson regression with robust error variance to individually regress exercise in the outcome wave (*t*_2_: 2014/2016) on baseline candidate predictors (at *t*_1_: 2010/2012) controlling for all covariates in the previous wave (*t*_0_: 2006/2008).

Results: Some physical health conditions (eg, physical functioning limitations and lung disease), psychological factors (eg, health mastery, purpose in life, and positive affect), and social factors (eg, helping others, religious service attendance, and volunteering) were robustly associated with increased subsequent exercise. Among factors related to psychological distress, perceived constraints stood out as a factor in reducing exercise.

Conclusions: We identified potentially novel exercise determinants, such as helping friends/neighbors/relatives, religious attendance, and volunteering, that have not been captured using a theory-driven approach. Future studies validating these findings experimentally in midlife and older adults are needed.

## Teaser

A nationwide data-driven investigation of retired Americans reveals that religious attendance, volunteering, and helping others are the strongest indicators of whether they exercise or not.

## Introduction

Numerous systematic reviews spanning various age groups consistently underscore the vital role of exercise in promoting healthy aging.^1–6^ Unfortunately, a progressive decline in physical activity is observed after age 45-50,^7,8^ a period in which the health benefits of exercise on aging become more pronounced considering the large increase in the incidence of preventable chronic diseases after age 45-50.^9^ This paradox is of paramount concern given the aging population. There are now 46.3 million Americans aged over 65, and this number is projected to increase by almost 50% in the next 15 years.^10^ Health organizations used to recommend 150-300 minutes of weekly moderate-to-vigorous physical activity until 2018, after which the guidelines eliminated the minimum duration for exercise sessions.^11–13^ The utility of engaging in at least some exercise was further corroborated by more recent robust epidemiological studies demonstrating that even brief bouts of exercise, lasting as little as 1 minute, can significantly reduce the risk of mortality and heart disease.^14,15^ Thus, assisting physically inactive older adults to start engaging in at least some regular exercise carries increasingly substantial public health significance.

For discerning the factors that elucidate exercise determinants, both induction and deduction serve as valuable epistemological tools.^16,17^ However, the empirical research of the past 4 decades has predominantly embraced a deductive methodology, anchored in the tenets of behavioral and social science theories. Notably, the investigation of exercise determinants began with atheoretical literature reviews,^18–20^ which evolved to the socioecological model, an expansive and multifaceted framework that extends beyond intra- and interindividual influences, such as the built environment and policy. Within the scope of intra- and interindividual determinants, behavioral science theories—initially focused on cognitive and social factors in the late 20th century, with a recent emphasis on affective determinants in the early 21st century—have proliferated a substantial body of empirical evidence clarifying why certain individuals refrain from engaging in exercise.^21–25^ While valuable for guiding our research, these theoretical models may constrict our investigative scope, inadvertently overlooking causal factors that do not fit neatly within established theoretical lenses. To complement this theoretical approach (ie, deduction), agnostically scrutinizing a large number of factors, including those not traditionally viewed as causal (ie, induction), may uncover new and unexpected insights. Such an approach may be particularly relevant for the putative causal factors that exert weak or no effect in younger age groups but becomes more salient as individuals reach middle age and older adulthood, a period characterized by physiological deterioration (eg, muscle loss and physical ailment) and shifts in social dynamics (eg, family structure) in which classic exercise determinants (eg, self-efficacy, social support, affect, and socioeconomic status) exert weaker or stronger effects on exercise participation.^26–28^

An innovative approach that may be able to identify novel targets of intervention is an exposure-wide approach, in which a large number of candidate predictors are examined with no a priori hypotheses,^29^ which can be refined to more principled approaches with respect to confounding control by focusing on exposures at a single point in time.^30,31^ We used such an exposure-wide approach to explore the determinants of changes in participation in at least some exercise (ie, from inactivity to some activity) among older US adults to help enrich the existing empirical literature in this area. We applied a lagged exposure-wide epidemiologic design, which is a data-driven approach aimed at elucidating promising predictors of a particular outcome that can then be examined further in subsequent research. This analytic approach includes steps that rigorously control for potential confounding and mitigate concerns about reverse causation,^30^ thereby strengthening causal inference. Using a nationwide sample of ~13 000 adults aged >50 years in the United States, we examined 62 candidate predictors of exercise participation drawn from the following domains: health behaviors, physical health, psychological well-being, psychological distress, social factors, and work. These predictors were selected to align with our prior studies using the same sample.^32–34^

## Methods

### Study population

We used data from the Health and Retirement Study (HRS), a nationwide panel study of people aged >50 in the United States.^35^ In 2006, HRS first assessed psychosocial data in a random 50% of participants who were selected to complete an enhanced face-to-face interview. The other 50% were assessed in the next wave (2008). After the interview, participants completed a psychosocial questionnaire which they then mailed back to the University of Michigan. The response rates were 88% in 2006 and 84% in 2008.^36^ Each HRS participant completes this psychosocial questionnaire every 4 years. Data from the 2006 and 2008 sub-cohorts were combined to increase sample size and statistical power. Participants were excluded if they did not report psychosocial data in this pre-baseline wave (since over half of the study predictors were psychosocial factors) resulting in a final sample of 13 771 participants.

We used data from 3 time points, each spaced 4 years apart: (1) covariates were assessed in the pre-baseline wave (*t*_0_: 2006/2008), (2) candidate predictors were assessed in the baseline wave (*t*_1_: 2010/2012), and (3) our outcome (ever exercise) was assessed in the outcome wave (*t*_2_: 2014/2016).

### Measures

#### Ever exercise

Exercise behavior was operationalized using the HRS item series denoting vigorous activity (vgactx) and moderate activity (mdactx). Participants indicated the frequency with which they engaged in vigorous (eg, running, swimming, and aerobics) and moderate (eg, gardening, dancing, and walking at a moderate pace) exercise over the past 12 months. For both vigorous and moderate activities, participants could select from 5 frequency options, including: (1) daily, (2) >1×/week, (3) 1×/week, (4) 1-3×/month, or (5) never. Given our focus on engaging in at least some exercise versus no exercise at all, this variable was dichotomized as never versus all else for each intensity. That is, those who reported engaging in either vigorous or moderate activities with a frequency ranging from daily to 1-3×/month were classified as “at least some exercisers.” Duration data were not available in the HRS dataset.

#### Covariates

We adjusted for a substantial number of covariates in the pre-baseline wave (*t*_0_: 2006/2008), including sociodemographics (age [continuous], gender [male/female], race/ethnicity [White, African-American, Hispanic, and Other], marital status [married/not married], income [<$50 000, $50 000-$74 999, $75 000-$99 999, and ≥$100 000], total wealth [based on quintiles of the score distribution for total wealth in this sample], educational attainment [no degree, GED/high school diploma, and ≥college degree], employment status [yes/no], health insurance [yes/no], geographic region [Northeast, Midwest, South, and West], religious service attendance [none, <1×/week, and ≥1×/week], personality [openness, conscientiousness, extraversion, agreeableness, and neuroticism; continuous], and childhood abuse [yes/no]). We adjusted for prior values of all predictors to evaluate change in each predictor.^30^ To reduce the possibility of reverse causation, we also adjusted for pre-baseline exercise.

We evaluated 62 candidate predictors in the baseline wave (*t*_1_: 2010/2012) including measures of: (1) health behaviors (ever exercise, smoking, heavy drinking, and sleep problems); (2) physical health (number of chronic conditions, diabetes, hypertension, stroke, cancer, heart disease, lung disease, arthritis, overweight/obesity, physical functioning limitations, cognitive impairment, chronic pain, self-rated health, hearing, and eyesight); (3) psychological well-being (positive affect, life satisfaction, optimism, purpose in life, mastery, health mastery, and financial mastery); (4) psychological distress (depression, depressive symptoms, hopelessness, negative affect, perceived constraints, anxiety, trait anger, state anger, cynical hostility, stressful life events, financial strain, daily discrimination, and major discrimination); (5) social factors (loneliness, living with spouse, frequency of contact in 3 separate relationship categories: (i) children, (ii) other family, and (iii) friends, closeness with spouse, number of close (i) children, (ii) other family, and (iii) friends, positive social support from (i) spouse, (ii) children, (iii) other family, and (iv) friends, negative social strain from (i) spouse, (ii) children, (iii) other family, and (iv) friends, volunteer activity, helping friends, neighbors, and relatives, religious service attendance, social status ladder ranking, and change in social status ladder ranking); and (6) employment (in the labor force). HRS materials provide further details about each variable.^37–39^

#### Multiple imputation

All missing exposures, covariates, and outcome variables were imputed using multiple imputations by chained equations; 5 imputed datasets were created. This method was used because it is more flexible than other methods of handling missing data and helps address problems that arise from attrition.^40–42^

### Statistical analysis

We used a lagged exposure-wide approach^30^ and ran separate models for each exposure. In our primary analyses, ever exercise was a binary outcome that was common (>10% in our sample). Thus, we used Poisson regression with robust error variance to individually regress exercise in the outcome wave (*t*_2_: 2014/2016) on baseline candidate predictors (at *t*_1_: 2010/2012), that is residualized changes in the likelihood of ever exercising 4 years later, after controlling for all covariates in the previous wave (*t*_0_: 2006/2008). Adjusting for pre-baseline levels of exposure helps us evaluate the association between “changes” in exposure and subsequent exercise behavior. As detailed in Appendix Text #1, this method isolates the impact of exposure changes over time on subsequent exercise outcomes. Because behavioral interventions target factors that drive changes in physical activity, rather than just predicting higher activity levels, analyzing changes in exposure is more relevant for developing effective interventions than simply evaluating exercise outcomes without accounting for baseline levels. Each predictor was examined one at a time in separate models. Continuous predictors were standardized (mean = 0, SD = 1) so their effect sizes could be interpreted as an SD change in the exposure. For categorical exposures, the effect estimate corresponds to associations between the exposure at baseline (at *t*_1_: 2010/2012), and ever exercising at the outcome wave (*t*_2_: 2014/2016), conditional on the exposure and covariates in the pre-baseline wave (at *t*_0_: 2006/2008). We marked multiple *P*-value cutoffs (including Bonferroni-corrected) and provided exact CIs, since multiple testing practices vary widely and are continuously evolving.^43,44^

#### Additional analyses

Unlike experimental studies with randomization, observational studies inherently provide weaker causal inferences regarding the hypothesized relationship between an exposure and an outcome. This limitation arises because it is impossible to completely rule out the influence of unmeasured confounders (ie, a covariate that has a causal influence on the independent variable and the outcome variable). Regardless of the size of the dataset, these unmeasured confounders can introduce bias. To evaluate the robustness of our results to potential unmeasured confounding, we calculated *E*-values to assess the minimum strength of unmeasured confounding on the risk ratio (RR) scale (with both the exposure and the outcome) needed to explain away the association between the exposure and outcome.^45^ As such, a higher *E*-value indicates that stronger unmeasured confounding would be required to overturn the association, suggesting greater confidence in the observed relationship. Conversely, a lower *E*-value suggests that even relatively weak unmeasured confounding could potentially alter the findings, highlighting the need for caution in interpreting the results.

## Results

**Figure 1.**
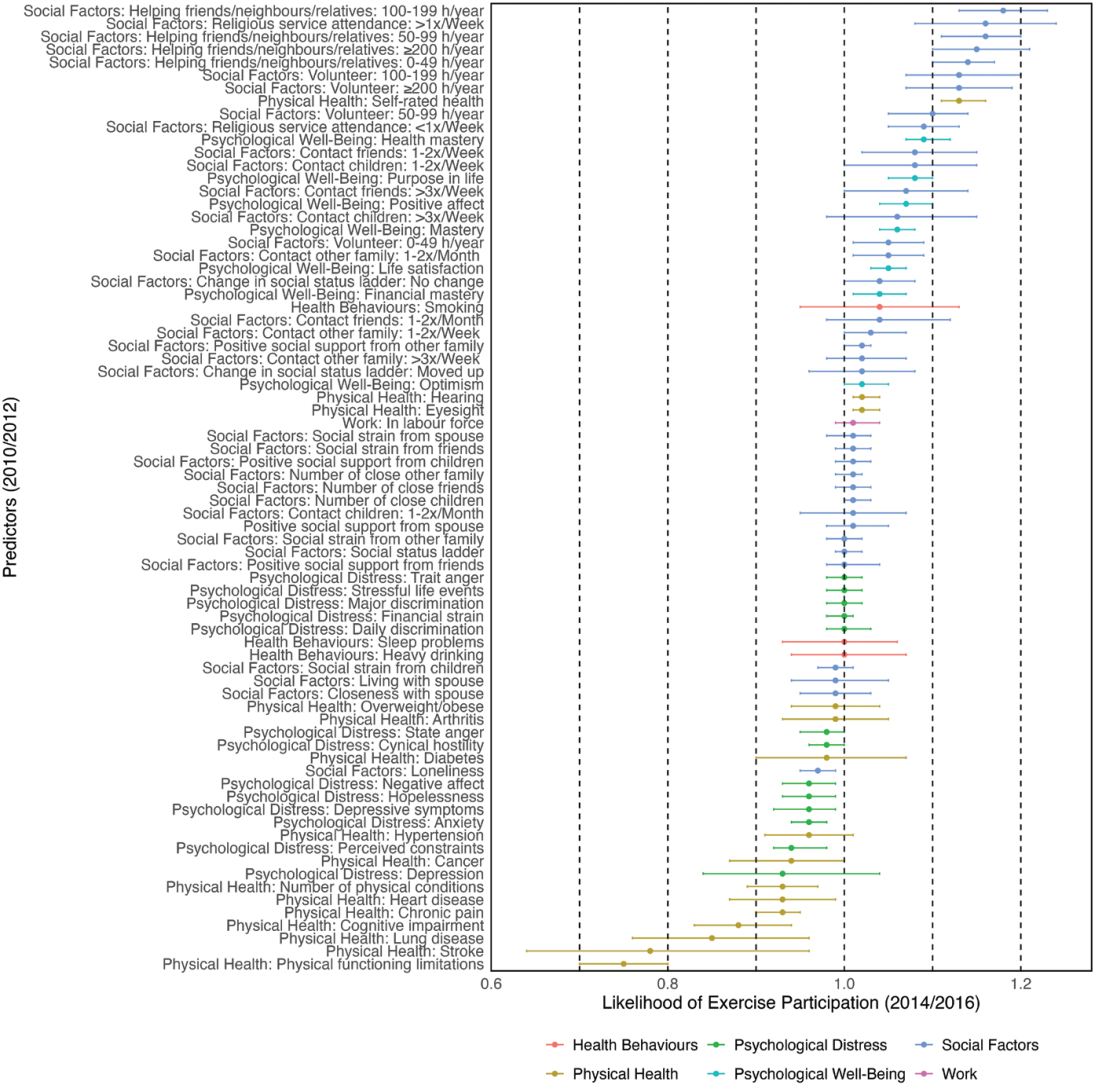
Likelihood of exercise participation based on candidate predictors. Values greater than 1 indicate a higher likelihood of participating in at least some exercise, and vice versa. CIs that include 1.0 indicate that a predictor is not statistically significant at the 5% level. Exercise was not included as a predictor (2010/2012), but they can be found in Table 2, which also includes a detailed explanation of the analyses.

In the pre-baseline wave (*t*_0_: 2006/2008), when all covariates were assessed, the average age of participants was 69 years old (SD = 10), more likely women (58%) and married (62%). Table 1 summarizes participant characteristics. Table 2 describes the changes in exercise from the pre-baseline wave (*t*_0_) to the outcome wave (*t*_2_). Among participants who reported ever exercising at *t*_0_, 75% continued to exercise at *t*_2_, while 25% no longer exercised. Conversely, of those who did not exercise at *t*_0_, 33% began exercising by *t*_2_, whereas 66% remained non-exercisers. Figure 1 and Table 3 show the associations between the candidate predictors and subsequent exercise.

**Table 1.** Characteristics of participants at pre-baseline (*N* = 13 757).^a^^,^^b^^,^^c^

Participant characteristics	No exercise (*n* = 2743)		Some exercise (*n* = 11 014)	
	No. (%)	Mean (SD)	No. (%)	Mean (SD)
Sociodemographic factors				
Age (y; range: 52-104)		72.7 (10.4)		68.4 (9.2)
Female (%)	1852 (67.5)		6183 (56.1)	
Race/ethnicity (%)				
White	1999 (72.88)		8632 (78.38)	
Black	443 (16.15)		1315 (11.94)	
Hispanic	251 (9.15)		837 (7.60)	
Other	50 (1.82)		229 (2.08)	
Married (%)	1408 (51.33)		7172 (65.12)	
Annual household income (%)				
<$50 000	2086 (76.05)		6268 (56.91)	
$50 000-$74 999	309 (11.27)		1814 (16.47)	
$75 000-$99 999	143 (5.21)		1032 (9.37)	
≥$100 000	205 (7.47)		1900 (17.25)	
Total wealth (%)				
1st quintile	912 (33.25)		1856 (16.85)	
2nd quintile	642 (23.41)		2098 (19.05)	
3rd quintile	534 (19.47)		2213 (20.09)	
4th quintile	348 (12.69)		2407 (21.85)	
5th quintile	307 (11.19)		2440 (22.15)	
Education (%)				
<High school	875 (31.93)		1838 (16.73)	
High school	1464 (53.43)		6039 (54.95)	
≥College	401 (14.64)		3112 (28.32)	
Employment				
In labor force	493 (17.97)		4285 (38.91)	
Health insurance (%)	2651 (96.68)		10 519 (95.57)	
Geographic region (%)				
Northeast	385 (14.08)		1706 (15.51)	
Midwest	700 (25.59)		2889 (26.27)	
South	1230 (44.97)		4262 (38.75)	
West	420 (15.36)		2142 (19.47)	
Childhood abuse (%)	166 (6.24)		682 (6.28)	
Health behaviors				
Frequent physical activity (%)	0 (0)		9869 (89.60)	
Smoking (%)	452 (16.56)		1272 (11.64)	
Heavy drinking (%)	119 (5.09)		672 (7.48)	
Sleep problems (%)	780 (54.97)		2272 (38.96)	
Physical health				
Number of physical conditions (range: 0-8)		3.29 (1.51)		2.46 (1.39)
Diabetes (%)	825 (30.08)		1897 (17.22)	
Hypertension (%)	1897 (69.16)		5941 (53.94)	
Stroke (%)	409 (14.91)		696 (6.32)	
Cancer (%)	497 (18.12)		1576 (14.31)	
Heart disease (%)	989 (36.06)		2362 (21.45)	
Lung disease (%)	476 (17.35)		822 (7.46)	
Arthritis (%)	1998 (72.84)		6285 (57.06)	
Overweight/obese (%)	1948 (72.63)		7534 (69.04)	
Physical functioning limitations (%)	1541 (56.18)		1786 (16.22)	
Cognitive impairment (%)	868 (32.59)		1833 (16.86)	
Chronic pain (%)	1405 (51.22)		3346 (30.39)	
Self-rated health (range: 1-5)		2.45 (1.07)		3.32 (1.02)
Hearing (range: 1-5)		3.09 (1.13)		3.37 (1.07)
Eyesight (range: 1-6)		3.82 (1.04)		4.27 (.959)
Psychological well-being				
Positive affect (range: 1-5)		3.28 (.80)		3.65 (.70)
Life satisfaction (range: 1-7)		4.55 (1.61)		5.15 (1.40)
Optimism (range: 1-6)		4.19 (.97)		4.53 (.94)
Purpose in life (range: 1-6)		4.15 (.97)		4.68 (.89)
Mastery (range: 1-6)		4.3 (1.21)		4.85 (1.06)
Health mastery (range: 0-10)		6.23 (2.82)		7.47 (2.20)
Financial mastery (range: 0-10)		6.83 (3.15)		7.46 (2.49)
Psychological distress				
Depression (%)	720 (27.05)		1160 (10.67)	
Depressive symptoms (range: 0-8)		2.37 (2.32)		1.16 (1.74)
Hopelessness (range: 1-6)		2.85 (1.39)		2.28 (1.24)
Negative affect (range: 1-5)		1.91 (.77)		1.62 (.59)
Perceived constraints (range: 1-6)		2.72 (1.32)		2.10 (1.13)
Anxiety (range: 1-4)		1.75 (.67)		1.52 (.55)
Trait anger (range: 1-4)		2.20 (.71)		2.15 (.66)
State anger (range: 1-4)		1.54 (.58)		1.48 (.49)
Cynical hostility (range: 1-6)		3.10 (1.16)		2.91 (1.12)
Stressful life events (range: 0-5)		.216 (.53)		.231 (.55)
Financial strain (range: 1-5)		2.15 (1.11)		1.91 (.96)
Daily discrimination (range: 1-6)		1.63 (.83)		1.62 (.71)
Major discrimination (range: 0-6)		.431 (.87)		.469 (.88)
Social factors				
Loneliness (range: 1-3)		1.62 (.60)		1.44 (.51)
Living with spouse/partner (%)	1427 (53.93)		7359 (68.65)	
Contact children (%)				
<very few months	401 (15.18)		1442 (13.41)	
1-2×/mo	280 (10.60)		1227 (11.41)	
1-2×/wk	693 (26.23)		3437 (31.97)	
≥3×/wk	1268 (47.99)		4646 (43.21)	
Contact other family (%)				
<Every few months	715 (26.88)		2552 (23.72)	
1-2×/mo	529 (19.89)		2592 (24.09)	
1-2×/wk	666 (25.04)		3011 (27.98)	
≥3×/wk	750 (28.20)		2606 (24.22)	
Contact friends (%)				
<Every few months	665 (24.87)		1600 (14.78)	
1-2×/mo	441 (16.49)		2037 (18.82)	
1-2×/wk	800 (29.92)		4007 (37.02)	
≥3×/wk	768 (28.72)		3179 (29.37)	
Closeness with spouse (range: 1-4)		3.36 (.83)		3.50 (.71)
Number of close children		2.94 (4.27)		2.77 (3.58)
Number of close other family		3.94 (4.99)		3.85 (5.69)
Number of close friends		4.04 (4.74)		4.65 (6.28)
Positive social support from spouse (range: 1-4)		3.33 (.74)		3.48 (.62)
Positive social support from children (range: 1-4)		3.23 (.77)		3.28 (.70)
Positive social support from other family (range: 1-4)		2.86 (.90)		2.89 (.86)
Positive social support from friends (range: 1-4)		2.99 (.78)		3.06 (.73)
Social strain from spouse (range: 1-4)		2.01 (.72)		1.9 (.66)
Social strain from children (range: 1-4)		1.74 (.69)		1.68 (.62)
Social strain from other family (range: 1-4)		1.58 (.65)		1.56 (.60)
Social strain from friends (range: 1-4)		1.82 (.46)		1.84 (.41)
Religious service attendance (%)				
Not at all	966 (35.26)		2485 (22.57)	
<1×/wk	787 (28.72)		3512 (31.90)	
≥1×/wk	987 (36.02)		5011 (45.52)	
Volunteering (%)				
0 h/y	2257 (82.34)		6660 (60.55)	
1-49 h/y	177 (6.46)		1352 (12.29)	
50-99 h/y	112 (4.09)		973 (8.85)	
100-199 h/y	105 (3.83)		1085 (9.86)	
≥200 h/y	90 (3.28)		930 (8.45)	
Helping friends/neighbors/relatives (%)				
0 h/y	2049 (74.84)		4573 (41.66)	
1-49 h/y	336 (12.27)		2875 (26.19)	
50-99 h/y	166 (6.06)		1669 (15.20)	
100-199 h/y	102 (3.73)		1087 (9.90)	
≥200 h/y	85 (3.10)		774 (7.05)	
Social status ladder (range: 1-10)		6.05 (1.94)		6.64 (1.68)
Change in social status ladder (%)				
Moved down	305 (11.84)		962 (9.04)	
No change	1987 (77.14)		8296 (77.94)	
Moved up	284 (11.02)		1386 (13.02)	
Personality				
Openness (range: 1-4)		2.77 (.58)		2.97 (.53)
Conscientiousness (range: 1-4)		3.19 (.53)		3.39 (.45)
Extraversion (range: 1-4)		3.01 (.59)		3.24 (.52)
Agreeableness (range: 1-4)		3.48 (.51)		3.53 (.46)
Neuroticism (range: 1-4)		2.16 (.66)		2.01 (.59)

^a^This table was created based on non-imputed data.

^b^All variables in this table were used as covariates, and assessed in the pre-baseline wave (*t*_0_: 2006/2008).

^c^The percentages in some sections may not add up to 100% due to rounding.

**Table 2.** Changes in ever exercising from the pre-baseline wave (*t*_0_) to the outcome wave (*t*_2_).^a^

Pre-baseline wave (*t*_0_)	Outcome wave (*t*_2_)	
	Ever exercise (%)	Do not ever exercise (%)
Ever exercise	75	25
Do not ever exercise	33	66

^a^The correlation coefficient for ever exercising between the pre-baseline wave (*t*_0_) and the outcome wave (*t*_2_) was 0.35.

**Table 3.** Candidate predictors of exercise (Health and Retirement Study [HRS]: *N* = 13 771).^a^^,^^b^^,^^c^

Candidate predictor	RR	95% CI
Health behaviors		
Ever exercise	1.86	1.73-2.01^***^
Smoking	1.04	0.95-1.13
Heavy drinking	1.00	0.94-1.07
Sleep problems	1.00	0.93-1.06
Physical health		
Number of physical conditions	0.93	0.89-0.97^**^
Diabetes	0.98	0.90-1.07
Hypertension	0.96	0.91-1.01
Stroke	0.78	0.64-0.96^*^
Cancer	0.94	0.87-1.00
Heart disease	0.93	0.87-0.99^*^
Lung disease	0.85	0.76-0.96^**^
Arthritis	0.99	0.93-1.05
Overweight/obese	0.99	0.94-1.04
Physical functioning limitations	0.75	0.70-0.80^***^
Cognitive impairment	0.88	0.83-0.94^**^
Chronic pain	0.93	0.90-0.95^***^
Self-rated health	1.13	1.11-1.16^***^
Hearing	1.02	1.01-1.04
Eyesight	1.02	1.01-1.04^**^
Psychological well-being		
Positive affect	1.07	1.04-1.10^***^
Life satisfaction	1.05	1.03-1.07^***^
Optimism	1.02	1.00-1.05
Purpose in life	1.08	1.05-1.10^***^
Mastery	1.06	1.04-1.08^***^
Health mastery	1.09	1.07-1.12^***^
Financial mastery	1.04	1.01-1.07^*^
Psychological distress		
Depression	0.93	0.84-1.04
Depressive symptoms	0.96	0.92-0.99^*^
Hopelessness	0.96	0.93-0.99^*^
Negative affect	0.96	0.93-0.99^**^
Perceived constraints	0.94	0.92-0.98^**^
Anxiety	0.96	0.94-0.98^**^
Trait anger	1.00	0.98-1.02
State anger	0.98	0.95-1.00
Cynical hostility	0.98	0.96-1.00
Stressful life events	1.00	0.98-1.02
Financial strain	1.00	0.98-1.01
Daily discrimination	1.00	0.98-1.03
Major discrimination	1.00	0.98-1.02
Social factors		
Loneliness	0.97	0.95-0.99^**^
Living with spouse	0.99	0.94-1.05
Contact children		
<Every few months	Reference	Reference
1-2×/mo	1.01	0.95-1.07
1-2×/wk	1.08 1211wdq	1.00-1.15^*^
≥3×/wk	1.06	0.98-1.15
Contact other family		
<Every few months	Reference	Reference
1-2×/mo	1.05	1.01-1.09^*^
1-2×/wk	1.03	1.00-1.07
≥3×/wk	1.02	0.98-1.07
Contact friends		
<Every few months	Reference	Reference
1-2×/mo	1.04	0.98-1.12
1-2×/wk	1.08	1.02-1.15^*^
≥3×/wk	1.07	1.00-1.14
Closeness with spouse	0.99	0.95-1.03
Number of close children	1.01	1.00-1.03
Number of close other family	1.01	0.99-1.02
Number of close friends	1.01	0.99-1.03
Positive social support from spouse	1.01	0.98-1.05
Positive social support from children	1.01	0.99-1.03
Positive social support from other family	1.02	1.00-1.03^*^
Positive social support from friends	1.00	0.98-1.04
Social strain from spouse	1.01	0.98-1.03
Social strain from children	0.99	0.97-1.01
Social strain from other family	1.00	0.98-1.02
Social strain from friends	1.01	0.99-1.03
Volunteer		
0 h/y	Reference	Reference
0-49 h/y	1.05	1.01-1.09^**^
50-99 h/y	1.10	1.05-1.14^***^
100-199 h/y	1.13	1.07-1.20^***^
≥200 h/y	1.13	1.07-1.19^***^
Helping friends/neighbors/relatives		
0 h/y	Reference	Reference
1-49 h/y	1.14	1.10-1.17^***^
50-99 h/y	1.16	1.11-1.20^***^
100-199 h/y	1.18	1.13-1.23^***^
≥200 h	1.15	1.10-1.21^***^
Religious service attendance		
Not at all	Reference	Reference
<1×/wk	1.09	1.05-1.13^***^
≥1×/wk	1.16	1.08-1.24^***^
Social status ladder	1.00	0.99-1.02
Change in social status ladder		
Moved down	Reference	Reference
No change	1.04	1.00-1.08^*^
Moved up	1.02	0.96-1.08
Work		
In labor force	1.01	0.99-1.04

Abbreviation: RR, risk ratio.

^a^The analytic sample was restricted to those who had participated in the pre-baseline wave (2006 or 2008). Multiple imputation was performed to impute missing data on the exposures, covariates, and outcomes. Candidate antecedents were assessed, one at a time, in wave 2 (2010/2012), and the outcome (ever exercise) was assessed in wave 3 (2014/2016). The following covariates were controlled for at wave 1 (2006/2008): sociodemographic characteristics (age, sex, race/ethnicity, marital status, income, total wealth, level of education, employment status, health insurance, and geographic region), childhood abuse, personality factors (openness, conscientiousness, extraversion, agreeableness, and neuroticism), and all of the predictor variables, including health behaviors (ever exercise, smoking, heavy drinking, and sleep problems), physical health (heart disease, cancer, stroke, arthritis, hypertension, overweight/obese, diabetes, lung disease, chronic pain, hearing, eyesight, self-rated health, physical functioning limitations, and cognitive impairment), social factors (living with spouse, frequency of contact with children, frequency of contact with other family, frequency of contact with friends, loneliness, closeness with spouse, number of close children, number of close other family, number of close friends, positive social support from spouse, positive social support from children, positive social support from friends, positive social support from other family, social strain from spouse, social strain from children, social strain from other family, social strain from friends, religious service attendance, volunteering, helping friends/neighbors/relatives, perceived social status, and change in perceived social status), psychological well-being factors (life satisfaction, positive affect, purpose in life, optimism, health mastery, financial mastery, and mastery), psychological distress (depressive symptoms, hopelessness, negative affect, perceived constraints, anxiety, trait anger, state anger, daily discrimination, major discrimination, cynical hostility, stressful life events, and financial strain), work (in labor force), and baseline values of the outcome (ever exercise).

^b^All continuous candidate antecedents were standardized (mean = 0; SD = 1).

^c^An exposure-wide analytic approach was used, and a separate model for each exposure was run.

^*^ *P* < .05 before Bonferroni correction;

^**^ *P* < .01 before Bonferroni correction;

^***^ *P* < .05 after Bonferroni correction (the *P* value cutoff for Bonferroni correction is *P* = .05/62 predictors = *P* < .0.00080645161).

When considering health behaviors, 1 out of 4 predictors were significantly associated with an increased likelihood of subsequent exercise. Not surprisingly, ever exercising in the past was associated with an increased likelihood of subsequent exercise with the highest effect size (RR = 1.86, 95% CI = 1.73-2.01). For physical health indicators, 9 out of 15 candidate predictors were associated with an increased likelihood of subsequent exercise. Among physical health indicators, physical functioning limitations (RR = 0.75, 95% CI = 0.70-0.80), stroke (RR *=* 0.78, 95% CI = 0.64-0.96), and lung disease (RR = 0.85, 95% CI = 0.76-0.96) were most strongly associated with decreased likelihood of subsequent exercise.

There was evidence that several psychological factors (11 out of 20 predictors) were associated with an increased likelihood of subsequent exercise. Among psychological well-being factors, health mastery (RR *=* 1.09, 95% CI = 1.07-1.12), purpose in life (RR = 1.08, 95% CI = 1.05-1.10), and positive affect (RR = 1.07, 95% CI = 1.04-1.10), were most strongly associated with increased likelihood of subsequent exercise. Among psychological distress factors, perceived constraints (RR = 0.94, 95% CI = 0.92-0.98) were most strongly associated with a lower likelihood of subsequent exercise; further depressive symptoms, hopelessness, negative affect, and anxiety all had RRs = 0.96 in relation to ever exercising. For social factors, there was notable evidence that 9 out of 22 predictors were associated with subsequent exercise. Helping friends/neighbors/relatives 100-199 hours/year (RR = 1.18, 95% CI = 1.13-1.23) and ≥200 hours/year (RR = 1.15, 95% CI = 1.10-1.21), religious service attendance (>1×/week) (RR = 1.16, 95% CI = 1.08-1.24), and volunteering 100-199 hours/year (RR = 1.13, 95% CI = 1.07-1.20) and ≥200 hours/year (RR *=* 1.13, 95% CI = 1.07-1.19) were most strongly associated with increased likelihood of subsequent exercise. Finally, being in the labor force was not notably associated with subsequent exercise.

### Additional analyses

*E*-values indicated that many of the observed associations were potentially moderately robust to unmeasured confounding (Table 4). For example, for purpose in life, an unmeasured confounder that was associated with both ever exercising and purpose in life by RRs of 1.36 each (above and beyond the covariates already adjusted for) could explain away the association, but weaker joint confounder associations could not. Furthermore, to shift the CI to include the null, an unmeasured confounder associated with both ever exercising and purpose in life by RRs of 1.25 each could explain away the association, but weaker joint confounder associations could not. This particular association is thus at least moderately robust to potential unmeasured confounding. However, in other cases, a combination of unmeasured confounding and statistical uncertainty might suffice to explain away the results.

**Table 4.** Robustness to unmeasured confounding (*E*-values) for the associations between candidate predictors and subsequent exercise (*N* = 13 771).^a^

	Effect estimate^b^	CI limit^c^
Health behaviors		
Frequent physical activity	3.13	2.81
Smoking	1.23	1
Heavy drinking	1.06	1
Sleep problems	1.07	1
Physical health		
Number of physical conditions	1.37	1.15
Diabetes	1.16	1
Hypertension	1.26	1
Stroke	1.87	1.19
Cancer	1.34	1
Heart disease	1.37	1
Lung disease	1.62	1.10
Arthritis	1.10	1
Overweight/obese	1.12	1
Physical functioning limitations	2.00	1.76
Cognitive impairment	1.52	1.26
Chronic pain	1.38	1.18
Self-rated health	1.52	1.43
Hearing	1.18	1
Eyesight	1.18	1
Psychological well-being		
Positive affect	1.35	1.23
Life satisfaction	1.28	1.15
Optimism	1.17	1
Purpose in life	1.36	1.25
Mastery	1.30	1.19
Health mastery	1.41	1.31
Financial mastery	1.23	1.06
Psychological distress		
Depression	1.34	1
Depressive symptoms	1.27	1.07
Hopelessness	1.24	1.05
Negative affect	1.25	1.08
Perceived constraints	1.30	1.15
Anxiety	1.25	1.12
Trait anger	1.03	1
State anger	1.18	1
Cynical hostility	1.14	1
Stressful life events	1.02	1
Financial strain	1.07	1
Daily discrimination	1.05	1
Major discrimination	1.05	1
Social factors		
Loneliness	1.20	1
Living with spouse	1.11	1
Contact children		
<Every few months	Reference	Reference
1-2×/mo	1.11	1
1-2×/wk	1.36	1
≥3×/wk	1.31	1
Contact other family		
<Every few months	Reference	Reference
1-2×/mo	1.28	1
1-2×/wk	1.22	1
≥3×/wk	1.18	1
Contact friends		
<Every few months	Reference	Reference
1-2×/mo	1.26	1
1-2×/wk	1.37	1
≥3×/wk	1.34	1
Closeness with spouse	1.11	1
Number of close children	1.14	1
Number of close other family	1.08	1
Number of close friends	1.11	1
Positive social support from spouse	1.12	1
Positive social support from children	1.12	1
Positive social support from other family	1.15	1
Positive social support from friends	1.10	1
Social strain from spouse	1.08	1
Social strain from children	1.10	1
Social strain from other family	1.09	1
Social strain from friends	1.04	1
Volunteer		
0 h/y	Reference	Reference
0-49 h/y	1.28	1
50-99 h/y	1.43	1.12
100-199 h/y	1.52	1.22
≥200 h/y	1.51	1.15
Helping friends/neighbors/relatives		
0 h/y	Reference	Reference
1-49 h/y	1.53	1.35
50-99 h/y	1.58	1.36
100-199 h/y	1.63	1.37
≥200 h	1.58	1.25
Religious service attendance		
Not at all	Reference	Reference
<×/wk	1.40	1.17
≥1×/wk	1.59	1.31
Social status ladder	1.07	1
Change in social status ladder		
Moved down	Reference	Reference
No change	1.25	1
Moved up	1.16	1
Work		
In labor force	1.11	1

^a^See VanderWeele and Ding^45^ for the formula for calculating *E*-values.

^b^The *E*-values for effect estimates are the minimum strength of association on the risk ratio scale that an unmeasured confounder would need to have with both the exposure and the outcome to fully explain away the observed association between the exposure and outcome, conditional on the measured covariates.

^c^The *E*-values for the limit of the 95% CI closest to the null denote the minimum strength of association on the risk ratio scale that an unmeasured confounder would need to have with both the exposure and the outcome to shift the CI to include the null value, conditional on the measured covariates.

## Discussion

In this study, we investigated putative determinants of engaging in at least some exercise among adults aged over 50 in the United States by examining 62 candidate predictors spanning health behaviors, physical health, psychological factors, social factors, and employment. Some factors were robustly associated with subsequent physical activity. The factors that most strongly predicted decreased likelihoods of subsequent exercise were physical functioning limitations, stroke, and lung disease. The factors that most strongly predicted an increased likelihood of subsequent exercise were religious service attendance, volunteering, and helping friends, neighbors, and relatives.

Our findings align with previous research, which has consistently demonstrated that exercise participation (eg, volume or frequency) is influenced by higher positive psychological well-being^46,47^ and social factors,^48^ as well as reduced psychological stress.^49^ Regarding potentially novel exercise determinants, it is worth highlighting that some of the eudemonic psychological well-being factors (eg, sense of purpose, volunteering, and religious service attendance) were associated with higher exercise levels. A predominant human inclination is to pursue pleasure and avoid pain as proposed by Epicurus, reiterated by Bentham, and empirically confirmed by Kahneman et al.^50,51^ The reluctance of most individuals to engage in exercise can be attributed to the fact that the experience of exercising is typically perceived as less pleasurable *during* (vs after) the activity.^24,52,53^ In this light, a common unmeasured factor that may increase exercise level—as well as purpose in life, volunteering, and religious activities—may be one’s willingness or behavioral tendency to engage in somatically unpleasant but meaningful activities. Potentially related, in our results, behavioral (vs psychological) phenotypes tend to show higher effect sizes. For instance, subfactors of psychological well-being predict exercise participation with small effect sizes (RR = 1.02-1.09), while behavioral phenotypes like helping others, volunteering, and religious attendance show effect sizes around RR = 1.09-1.18. Older adults who have lower neural conflict when making eudaimonically (vs hedonically) good decisions may succeed in practicing their ideas (eg, knowledge and beliefs) behaviorally. For example, in a functional magnetic resonance imaging study with 220 sedentary and overweight young adults, those with a higher sense of purpose had reduced cognitive conflict when exposed to health messages about physical activity.^54^ It is important to note that eudemonic psychological well-being and exercise appear to have a reciprocal relationship,^55,56^ and unmeasured third variables could account for some of the relations between eudaimonic psychological well-being and physical activity. A prior randomized controlled experiment has shown that those whose gym attendance was incentivized by charitable donations on their behalf made 12 additional visits to the YMCA over a month, compared with those in the control condition who only received reminder emails about their gym attendance.^57^ This suggests that enhancing meaning and purpose may have a causal impact on enhanced exercise behavior.

It is important to emphasize that our exposure-wide approach aims to generate hypotheses rather than conduct formal hypothesis testing. This method has proven highly successful in human genetics, particularly in the context of genome-wide association studies, leading to transformative advancements in the past decade. Notably, because one’s genetic profile is determined before all behavioral phenotypes, genetics enjoys an advantage of aligned temporality between exposure and outcome. Moreover, except for ancestral history, there is no conceptual confounder that can have a causal influence on both genotype (ie, allele frequency within a population) and phenotype. However, in non-genetic research, these conditions are seldom met.^31^ Thus, despite our efforts to mitigate confounding through robust covariate adjustment, longitudinal design, and *E*-value analyses, our exposure-wide results should be considered hypothesis-generating. Therefore, newly identified factors warrant further investigation in studies providing stronger causal inferences (eg, randomized controlled trials); and then, studies examining the feasibility of engaging the intervention target.^58^ Finally, an exposure-wide approach for understanding exercise determinants can be applied to other populations with different sociodemographic backgrounds (eg, age and country).

One crucial aspect often overlooked within our theoretical framework of exercise determinants is health condition. Physical and mental capabilities are posited as an influence in some models of behavior change such as COM-B model (Capability, Motivation, Opportunity-Behavior),^59^ but capabilities are not addressed in widely used explanatory theoretical models of health behavior motivation. Indeed, we found that medical conditions, such as physical functioning limitations, stroke, and lung disease were strong determinants of subsequent exercise behavior. As the global population of older adults continues to grow, an increasing number of individuals will be grappling with a spectrum of underlying health issues. This demographic shift underscores the escalating need to place greater emphasis on tertiary prevention efforts among older populations who tend to have underlying illnesses. For example, studies aimed at enhancing exercise levels after treating underlying illnesses using pharmaceutical or surgical treatment may become increasingly pertinent. Future studies may aim to integrate treatment with subsequent rehabilitation efforts, such as physical therapy and personal training, particularly for older adults. This approach not only promotes physical recovery but also improves overall health outcomes by ensuring that treatment transitions seamlessly into effective rehabilitation. Additionally, exploring innovative strategies to integrate exercise promotion programs into hospitals and rehabilitation programs for older adults may be a promising avenue for maximizing the utility of exercise for the population’s health. For instance, wearable technologies and AI-driven systems offer the potential for highly personalized exercise regimens for the elderly, going beyond biological factors (eg, age, weight, and blood pressure) to consider older adults’ behavioral patterns (eg, hospital visit schedule, mood fluctuations, daily routines, and seasonal variations).^60–62^ Connecting these technologies to the hospital’s web portal may enrich the physician–patient experience and ultimately improve tertiary prevention efforts among older populations with underlying illnesses.

All the effect sizes in this study are considered small. For reference, in epidemiologic studies, relative risk values of 1.22, 1.86, and 3.00 are interpreted as small, medium, and large effect sizes, respectively.^63^ Moreover, exercise participation was influenced by a multitude of factors, each with modest effects, in line with a common pattern observed in other exposure-wide approaches, such as genome association studies. Moreover, in line with the socioecological model, our findings ascertained that exercise behavior in older adults is influenced by numerous determinants, highlighting the complexity of this relationship. Relatedly, recent advances in poly-exposure scores, which combine numerous small effects of each exposure into a cumulative score, may enable researchers to more effectively and confidently identify subpopulations predisposed to low levels of exercise, especially when these scores include factors that are difficult or impossible to change, such as genetics and the built environment. While, poly-approaches, such as poly-exposure scores or genetic risk scores, are effective in *predicting* behaviors, they have limited utility for *changing* behaviors. Thus, the factors that are fixed or unmodifiable may be used to identify high-risk populations for targeted interventions, while modifiable factors may be further examined as the target of an intervention. Future intervention studies aimed at changing behaviors among high-risk populations based on a poly-exposure score approach could explore strategies for integrating multiple intervention targets within a single trial. Such interventions may encompass the targeting of psychological and social determinants, the attenuation of risk factors (eg, depression), the enhancement of health assets (eg, social engagement and volunteering), and the integration of pharmaceutical treatments. Notably, advanced intervention frameworks such as Multiphase Optimization Strategy^64^ and Behavior Change Taxonomy^65^ may serve as effective guidance for translating big-data-driven poly-exposure approaches into personalized behavioral interventions.

Our study has some limitations. First, exercise phenotype was self-reported, which is susceptible to bias. While the accuracy may be low in assessing the exact volume of exercise, it is likely accurate in distinguishing whether someone engages in exercise or not. Relatedly, we lacked a measure of duration, which is expected to vary across individuals who exercise, and the factors that distinguish no activity from some activity may not be the same as those that predict activity duration. Second, our study did not address how predictors of exercise differ by key factors, such as age, gender, or socioeconomic status that often modify exposure’s effect on physical activity.^26,66^ Third, although the analyses were conducted systematically using an exposure-wide approach, the selection of candidate predictors, covariates, outcome, and their operationalizations was informed by our expertise in social epidemiology, exercise science, and behavioral medicine, thereby introducing a degree of subjectivity. Related, the present study was not pre-registered. However, our team has consistently published exposure-wide studies using nearly identical predictors and covariates.^32–34^ Fourth, while we assumed that covariates measured at pre-baseline would remain stable, this assumption may not be warranted, and could affect the effect sizes observed. Finally, each exposure examined can be correlated. Despite potential collinearity, we intentionally analyzed individual exposures separately (while controlling for numerous other potential confounders in the prior wave) as we sought to understand each factor’s independent contribution, aligning with the goal of generating hypotheses for potential intervention in exposure-wide approaches. However, future research aimed at building a predictive model may incorporate additional multivariate or machine learning methods, such as random forest or lasso regression, to further address variable correlations and refine predictor selection.^67–70^ Finally, the majority of HRS participants were White. Future research should validate these predictors in a more diverse population and incorporate additional exposures related to social determinants of health, such as food and housing insecurity, neighborhood safety, and financial stress. The current study also has several notable strengths. First, to our knowledge, this is the first exposure-wide study to examine determinants of exercise behavior. We investigated many novel predictors that are understudied or not previously studied, and also evaluated all predictors within the same study, allowing us to compare effect sizes. Second, our study has high generalizability from using a nationwide dataset of older adults in the United States.

## Conclusion

Using an exposure-wide approach in a prospective cohort study of older US adults, we have identified a critical oversight within our theoretical framework of exercise determinants: physical health conditions, such as stroke and lung disease. Given the growing population of older adults, it is prudent for funding agencies to allocate increased resources for tertiary prevention related to exercise. Furthermore, academic societies may foster discussions on tertiary prevention initiatives, particularly in the context of exercise, to stimulate greater research endeavors aimed at promoting exercise among older adults with health challenges.

We have identified potentially novel exercise determinants that do not align neatly with existing theories of exercise behavior, namely helping friends/neighbors/relatives, volunteering, religious attendance, and purpose in life. A common underlying factor that enables exercise participation as well as these eudemonic elements may be one’s willingness or behavioral tendency to engage in somatically unpleasant but meaningful activities. Putting this observation into a broader context, modern humans enjoy unprecedented somatic comfort (eg, entertainment at our fingertips, modern climate control technologies, labor-saving household appliances, online shopping, and food delivery services), thanks to scientific advancements and technological innovations, with even more advancements on the horizon. With this prospect, the significance of eudemonic elements and their impact on public health might gain greater importance.

Finally, while the aforementioned eudemonic elements need to be further scrutinized with study designs with stronger causal inferences, we demonstrate the potential of the exposure-wide approach for uncovering novel determinants of exercise behavior within specific populations. Thus, adopting similar exposure-wide methodologies in diverse large-scale cohorts with varying sociodemographic characteristics holds promise for identifying additional yet unexplored factors influencing exercise behavior. In translating exposure-wide studies to intervention development, the factors that are fixed or unmodifiable may be used to identify high-risk populations for targeted interventions, while modifiable factors may be further examined as the target of an intervention. Such a data-driven approach could further refine existing health behavior theories, similar to how theoretical research in exercise motivation in the past later contributed to the development of the socioecological framework. In particular, considering the contemporary landscape of behavioral and social sciences, in which most research participants are Eurocentric and over-represent well-educated, high-income people on a global scale,^71^ nationally representative survey data, especially from non-Western countries, can make valuable contributions to theoretical frameworks in behavioral and social sciences. Future exposure-wide studies in more diverse and representative samples will enhance the generalizability of findings and can lead to a more nuanced refinement of theories of human behavior across different cultural and socioeconomic contexts worldwide.

## Data Availability

Data are publicly available through the Health and Retirement Study (http://hrsonline.isr.umich.edu/). Documentation, code, and other materials are available upon request.

## Study registration

This study was not formally registered.

## Analytic plan pre-registration

The analysis plan was not formally pre-registered.

## Appendix Text 1

### Proof illustrating how adjusting for pre-baseline levels of exposure can help us evaluate how “changes” in exposure are associated with subsequent ever exercising over time

Let *Y* be the ever exercising outcome in 2014/2016, *A*_1_ the exposure under consideration in 2010/2012, *A*_0_ the prior level of exposure in 2006/2008, and *C* the set of all other covariates in 2006/2008.

For a binary outcome, the regression model is: $log\left\{P\left[Y=1|a_{0},a_{1},c\right]\right\}=v+b_{0}a_{0}+b_{1}a_{1}+{b^{\prime }}_{2}c,$ which, if the outcome is rare can be approximated by a logistic regression model, $logit\left\{P\left[Y=1|a_{0},a_{1},c\right]\right\}=v+b_{0}a_{0}+b_{1}a_{1}+{b^{\prime }}_{2}c$, or, if the outcome is common, by a modified Poisson model. Let *Y*_*a*_ denote the potential outcome *Y* for an individual under an intervention to set *A*_1_ to *a*. For an individual with baseline exposure *A*_0_ = *a*_0_ and covariates *c* in 2006/2008, under the no-confounding (and positivity and consistency) and modeling assumptions, a change in exposure of *d* points *A*_0_ = *a*_0_ to *A*_1_ = *a*_0_ + *d* in 2010/2012, rather than maintaining exposure of *A*_1_ = *a*_0_ in 2010/2012, will give rise to an effect on the risk ratio scale of:

$$\begin{array}{l} \begin{array}{lll} & P\left[Y_{a0+d}=1|A_{0}=a_{0},c\right]/P\left[Y_{a0}=1|A_{0}=a_{0},c\right]\; & \begin{array}{l} =P\left[Y_{a0+d}=1|A_{1}=a_{0}+d,A_{0}=a_{0},c\right]/P\left[Y_{a0}=1|A_{1}=a_{0},A_{0}=a_{0},c\right]\;=P\left[Y=1|A_{1}=a_{0}+d,A_{0}=a_{0},c\right]/P\left[Y=1|A_{1}=a_{0},A_{0}=a_{0},c\right]\;=exp\left[v+b_{0}a_{0}+b_{1}\left(a_{0}+d\right)+{b^{\prime }}_{2}c\right]/exp\left[v+b_{0}a_{0}+b_{1}a_{0}+{b^{\prime }}_{2}c\right]\;=exp\left(b_{1}d\right)\; \end{array}\; \end{array} \end{array}$$

where the first equality follows by the no-confounding assumption, the second by consistency, and the third by the statistical model.
